# Supplementary material for: Are carbon emissions trading and green financial instruments synergistic? -Comprehensive quantitative research based on content analysis
Source: PLoS One. 2024 Mar 7;19(3):e0298601. doi: 10.1371/journal.pone.0298601 (PMC10919601; doi:10.1371/journal.pone.0298601)
Supplement: S1 Data — (ZIP) [file pone.0298601.s001.zip › Data/地级市Z_F工作报告.zip!/╕╩╦α/┬ñ─╧╩╨/└┤╫╘╣1⁄2╓┌║┼í╛┬φ┐╦╩2╛▌═°í┐_2011.docx]

各位代表：

现在，我代表市人民政府向大会作政府工作报告，请予审议，并请各位政协委员和列席人员提出意见。

“十一五”规划完成情况及2010年工作回顾

“十一五”时期是我市发展进程中极不平凡的五年。面对改革发展稳定的繁重任务、“5.12”特大地震的严重影响和国际金融危机的冲击，我们在省委、省政府和市委的坚强领导下，坚定信心，攻坚克难，开拓进取，埋头苦干，奋力推进科学发展和灾后重建，提前一年完成了“十一五”规划经济社会发展主要目标任务，顺利实现了地震灾后恢复重建三年任务两年基本完成的目标，为进一步加快发展打下了坚实的基础。

——结构调整初见成效，经济总量不断扩大。“十一五”末，全市生产总值（GDP）预计达到160亿元，年均增长10.5%；人均GDP达到5982元，年均增长11.4%。三次产业结构由“十五”末的32:29:39调整为25:24:51。全社会固定资产投资达到243亿元，年均增长52.8%，其中城镇500万元以上固定资产投资达到167亿元。大口径财政收入达到20.65亿元，年均增长25.5%；市县级财政收入达到9.33亿元，年均增长28.2%；财政支出达到137.1亿元，年均增长42.3%。全社会消费品零售总额达到41.5亿元，年均增长16.4%。城镇居民人均可支配收入达到10570元，年均增长10.9 %。农民人均纯收入达到2236元，年均增长13.6 %。

——着力推进科学重建，灾后重建任务基本完成。“5.12”特大地震发生后，我们在党中央、国务院和省委、省政府的坚强领导和关心支持下，以最快的速度开展应急抢险、群众生活安置、灾区生产生活秩序恢复和灾后恢复重建工作，最大限度地减少了人民群众生命财产损失，基本完成了灾后重建任务。全市累计开工地震灾后恢复重建项目3761项（不含城乡住房），占已落实资金项目3941项的95.4%；竣工项目2942项，占重建项目总数的74.7%，累计完成投资315亿元，占已落实资金重建项目总投资385.5亿元的81.7%。农村居民住房恢复重建全面完成并通过了市级验收；城镇居民住房重建基本完成；教育、卫生等社会事业项目基本实现灾后重建目标任务；基础设施、农村建设、城镇体系、生产力布局和产业调整、市场服务体系、生态环境、防灾减灾、精神家园等重建项目取得新进展。深圳市直接实施的20个援建项目已全部竣工并整体移交。

——农村经济持续发展，农民收入较快增长。五年来，财政对农业的投入达到42亿元，新增有效灌溉面积18.6万亩，新修梯田53万亩，粮食产量连续四年丰收，稳定在90万吨以上；农业特色产业基地达到868万亩，累计建成市级以上重点龙头企业47户。完成退耕还林105.55万亩，天然林保护1013.7万亩，治理水土流失1703平方公里，全市森林覆盖率由38.9%提高到42.5%。发放各种惠农补贴资金51亿元，农民人均达到206.5元；农村贫困人口由“十五”末的89.7万人减少到69万人，贫困发生率下降10个百分点；累计输转农村劳动力312.94万人次，创劳务收入171.59亿元，年均分别增长5.67%和31.98%。

——工业项目建设成就突出，综合实力不断增强。五年来，全市工业项目建设累计完成投资136亿元，建成投资千万元以上的重大工业项目105个，其中投资亿元以上的9个，成州锌业公司10万吨锌冶炼、独一味二三期扩建、成县120万吨和武都100万吨干法水泥、麒麟寺水电站、汉坪嘴水电站、金徽酒业万吨白酒生产线扩建等一批大项目相继建成投产。成州集团、洛坝集团、甘肃独一味生物制药有限公司跻身甘肃工业100强。万元生产总值能耗比“十五”末降低20%，二氧化硫、化学需氧量排放得到有效控制，实现了“十一五”节能减排目标。全市规模以上工业增加值由14.1亿元增加到23.5亿元，年均增长10.4%。

——固定资产投资快速增长，基础设施条件明显改善。五年累计完成固定资产投资647.7亿元，是“十五”时期的6倍。兰渝铁路、武罐高速公路等重大项目建设进展顺利，国省干线公路灾后恢复重建全面完成。建成通乡油路71条1546公里、通乡等级公路35条448公里、通村公路2637条13379公里。农网改造二期工程全面完成，新增发电装机容量22.5万千瓦，行政村和农户通电率分别达到100%和99.92%。移动电话、固定电话、宽带用户分别达到128.45万户、21.02万户和5.36万户。市区开发改造和八县县城、重点镇建设取得新的进展，全市城镇规划控制面积增加到140平方公里，建成区面积由32.43平方公里增加到49.98平方公里，城镇化率达到22.6%，比“十五”末提高10.71个百分点。

——旅游开发态势良好，第三产业发展步伐加快。第三产业完成增加值81亿元，年均增长14.9%，占到生产总值的50.6%。全市旅游发展总体规划通过省级评审，编制完成宕昌官鹅沟等11个景区的修建性详规；创建了官鹅沟、阳坝、万象洞、西狭颂4个国家4A级景区和3个2A级景区，旅游星级饭店达到19家，初步形成了工业、农业、乡村、体育旅游等新型旅游体系，旅游知名度和影响力不断提高。“十一五”期间累计接待游客980.97万人次，年均增长18.48%;实现旅游综合收入239.5亿元，年均增长32.15%。市场体系建设稳步推进，建成了一批有一定规模的专业市场，推动了商贸物流和服务业的发展。

——民生保障工作不断加强，社会事业取得新的进步。五年来，共投入资金63.52亿元，实施各类社会事业项目1793个，有力地促进了社会事业发展。城乡办学条件明显改善，实现城乡九年免费义务教育，全面完成“两基”攻坚目标，中等职业教育在校人数达到2.2万人，陇南师专顺利通过教育部人才培养工作水平评估。基层卫生服务体系建设不断完善，乡镇卫生院职工工资实现由财政全部供给，新型农村合作医疗保障覆盖面扩大，参合率达到96.61%。新建了一批文化、广电、体育基础设施，广播电视村村通、乡镇文化站、文化大院、农家书屋建设顺利推进，全民健身活动广泛开展。完成科技培训215万人次，实施各类科技项目233项，取得科技成果123项，获得省级科技进步三等奖3项，申请专利281件。人口和计划生育服务体系基本建成，人口自然增长率控制在6.25‰以下。新增城镇就业岗位6.42万个，城镇登记失业率控制在4%以内。城市低保覆盖面不断扩大，农村低保、医疗救助等新型社会救助政策顺利实施，弱势群体和低收入人群基本生活得到有效保障。地震监测、气象、地质灾害预警预报信息平台体系基本建成，防灾减灾能力不断增强。国防教育、人民防空、民兵预备役建设、“双拥”工作不断深入。外事侨务、统计、残疾人事业、档案等各项工作都取得了新进步。

——改革开放不断深化，发展活力明显增强。市县政府机构改革基本完成，行政审批制度改革不断深化，行政审批和许可事项减少349项；国有企业改革基本完成，改制企业184户；农村改革稳步推进，集体林权制度改革全面推开，确权面积达到1118.16万亩；财政管理体制改革不断完善，部门预算编制、强农惠农资金发放、政府采购等改革措施全面实行。对外开放扩大，五年累计完成进出口总额4241万美元；签约各类招商引资项目663项，累计到位资金157.39亿元。非公经济快速发展，占生产总值比重达到44.7%，比“十五”末增加5.7个百分点。

——民主法制建设取得实效，社会大局保持稳定。依法行政工作得到加强，对涉及政府决策的重大事项实行了公示和听证制度，政务公开和政府信息公开制度全面实施，制定发布规范性文件79件，实行严格的行政执法主体资格制度，依法办理行政复议案件。认真执行市人大及其常委会的各项决议、决定，主动接受市人大的工作监督、市政协的民主监督，办理答复人大代表建议1016件、政协委员提案1311件，办复率100%。认真落实廉政建设责任制，强化审计监督和行政监察，查处了一批违法违纪案件。信访、安全生产、应急管理、社会治安综合治理工作得到加强，保持了社会大局稳定。

各位代表，刚刚过去的2010年，我们认真贯彻落实省委、省政府和市委的决策部署，紧紧围绕灾后恢复重建和经济社会发展工作任务，全力以赴抓落实，着力在重点上推进，难点上突破，完成了市二届五次人代会确定的各项目标任务。与2009年相比，预计生产总值增长10.2%，全社会固定资产投资增长21%（其中城镇500万元以上固定资产投资增长35.3%），社会消费品零售总额增长17%，大口径财政收入增长20.9%，市县级财政收入增长17.5%，城镇居民人均可支配收入增长11.5%，农民人均纯收入增长12.1 %，城镇登记失业率为3.8%。一年来，主要做了九个方面的工作：

一是灾后重建取得新成绩。在上年全面完成农村居民住房重建的基础上，我们及时将灾后重建的重点转移到城镇居民住房重建和公共服务、基础设施恢复重建上来，着力解决阶段性重要问题，建立健全未开工项目领导包抓责任制，加快项目审批进度，强化督查考核和验收，有力地促进了项目建设。当年新开工各类项目1968项，竣工2480项，完成投资118.1亿元，其中教育、卫生项目新开工251项、竣工545项，基本实现年度目标任务。合理调整了城镇居民住房维修重建规划基数，城镇居民住房维修重建当年新开工54430户，竣工53420户，开工率、竣工率分别达到100%和97.4%。加强项目整合，合理调整资金使用属性，有效提高了重建资金使用效益。

二是项目建设实现新突破。全年争取国家和省上投资9.51亿元，新开工市列重点建设项目155项，预计完成投资104.3亿元。兰渝铁路、武罐高速公路累计完成投资69.5亿元，成武高速公路开工建设，十天高速公路陇南段、成州民用机场等重大项目前期工作取得新进展。文县临江330千伏、武都东江、文县石坊等110千伏送变电工程项目建设进展顺利。实施农田水利项目118项，新增水浇地3.69万亩、集雨补灌4.05万亩、节水灌溉5.2万亩。完成生态林建设20万亩、公路绿化美化400公里，治理水土流失410平方公里。加大招商引资力度，当年签约招商引资合同项目106个，签约金额94.53亿元，到位资金43.85亿元。

三是“三农”工作取得新进展。加大了“三农”投入力度，设立1000万元市级特色农业发展基金，争取农业基本建设投入1.55亿元，实施了一批农业特色产业、土地复垦整理项目，全年新增特色产业面积80.95万亩，新增梯田17.01万亩、耕地8310亩。特色产业龙头企业、市场建设、农民专业合作经济组织、标准化生产都取得了新进展。推广全膜双垄沟播35.4万亩，粮食总产达到97.9万吨。实施扶贫整村推进100个，全面完成“5.12”地震异地搬迁安置任务，培训农民工35.47万人，输转农村劳动力69.65万人，创劳务收入56.69亿元，同比增长23.8%。

四是工业发展迈出新步伐。全年实施市列重点工业项目41项，厂坝铅锌矿日处理4500吨矿石选矿系统技改、两当招金矿业公司日采选1000吨原矿等19个项目建成投产，累计完成投资16.5亿元。认真落实扶持企业发展的各项优惠政策，市列工业发展基金1500万元，其中安排1000万元贴息资金，支持成县、徽县“8.12”重灾区企业恢复生产。实施工业灾后重建项目43个，完成投资25.3亿元，其中已建成18个。成县厂坝矿区资源整合与资产重组工作基本完成，新公司即将挂牌运营。西成经济开发区已完成扩区总体规划，陇南徽县工业集中区发展规划通过省级批复。充分发挥工业领导小组的作用，加大协调服务力度，及时研究解决工业经济运行中存在的困难和问题，促进了工业经济稳定增长，工业增加值同比增长18.5%。

五是城镇建设呈现新面貌。陇南市总体规划和各县总体规划编制工作进展顺利，投入1885万元，启动了市区汉王、两水、吉石坝组团和部分县城控制性详细规划编制工作，部分县及重点镇总体规划修编、编制工作正在开展。加强对市区东江新区和其它县城沿江沿河两岸、主干道临街建筑的界面、形态、色彩设计，强化建筑管理，提升了城市建设水平。全年实施城市建设项目162项，总投资24.03亿元，完成投资10.89亿元。市区和各县县城新区开发、旧城改造及重点镇建设取得新的成效，规模进一步扩大，功能进一步完善，城市面貌发生了明显变化。

六是第三产业有了新发展。加大旅游投入，设立了市级旅游发展专项资金，建成旅游基础设施建设项目13个，完成投资7400万元，旅游服务质量得到加强。预计全年接待游客320万人次，实现旅游综合收入8.5亿元，分别比上年增长22.9%和57.4%。扎实推进“万村千乡市场工程”、“新网工程”建设，建成各类市场67个。“家电下乡”、“汽车以旧换新”完成销售1.8亿元，完成进出口贸易总额445万美元。

七是社会事业发展取得新成效。全年投入资金2.12亿元，有力地促进了社会事业的发展。深入开展教育质量提高年活动，推进教育内涵式发展，高考二本以上上线人数达到2484人，比上年增加965人，建成中小学校舍安全工程49所。积极开展基本医药卫生制度改革，基层卫生院（所）基本药物零差率销售全面推进。建成乡镇综合文化站25个、在建169个，建成农家书屋642家、文化大院29个、农村文化资源信息共享工程941个。下派科技特派员1313名，申报省级科技计划项目78项，申请专利49件。农村计划生育率达到88.21%，人口出生率控制在13.2‰以下。新增养老保险17074人、失业保险2557人、城镇职工医疗保险27525人、工伤保险3637人，累计发放临时救助资金和城乡医疗救助资金912万元、3128万元。

八是民生工作取得新进步。市委、市政府向群众承诺的13件实事全面完成，新修通村等级公路219条1085公里，建成便民桥92座；建成农村饮水安全工程141项，解决了14.71万农村人口的饮水安全问题；实施扶贫整村推进100个，预计减少贫困人口7.87万人；配备太阳灶2.8万台；发放租赁住房补贴5634户15243人，建设廉租住房1864套9.32万平方米，经济适用房2366套21.49万平方米,城市和国有林区、工矿棚户区改造2546户；新增城镇就业1.5万人，公开招考高校毕业生就业4312人；全市城镇职工基本医疗保险最高支付限额由2万元提高到4万元；新农合补助标准由80元提高到120元；给78所乡镇卫生院配备了救护车；建成“五保”家园432所，实施了困难群众临时生活救助政策；改造提升食品加工小作坊200个；深入开展了交通秩序、市场秩序、城乡环境卫生整治和文明长廊建设活动，九县区垃圾处理场全部建成；对国、省道沿线容易出现占道经营的40个集贸市场集中进行综合整治。有效解决了部分群众行路、住房、上学、就业、就医和特殊困难群体生活等问题。

九是抗洪救灾取得全面胜利。“8.12”特大暴洪灾害发生后，我们在第一时间启动抢险应急预案，迅速组织广大干部群众投入抗洪抢险救灾，营救被困群众，救治受伤人员，安置群众生活，紧急抢修交通、电力、通讯等基础设施，扎实开展卫生防疫工作，严密防范次生灾害，确保了灾区人心安定、社会稳定，夺取了抗洪抢险工作的全面胜利。驻陇南解放军、武警部队和民兵预备役官兵第一时间投入抢险救灾，发挥了主力军作用。认真开展灾情核查评估和灾后重建规划编制，积极争取国家、省上灾后重建政策支持，省政府已出台《支持成县8.12暴洪灾害恢复重建工作实施方案》，安排项目资金8.46亿元，重建工作有序开展。

各位代表，过去的五年，在困难和挑战面前，我们始终坚持以科学发展观统领经济社会发展全局，紧紧抓住发展第一要务不动摇，迎难而上，开拓进取，不仅经受住了严峻考验，掌握了经济工作的主动权，同时也为今后工作特别是驾驭复杂局面、促进经济社会跨越发展积累了宝贵经验。回顾“十一五”时期特别是近两年来的工作，我们感到有七个方面的突出特点：一是把握大局，与时俱进，推动科学发展的思路不断创新完善。市委坚持把科学发展观的根本要求与陇南实际相结合，在深化市情认识中把握发展规律和阶段性特征，不断拓展完善科学发展的总体思路，在交通建设、工业发展、城镇建设、农业特色产业、旅游产业开发、教育事业发展等方面提出了一系列具体要求，使工作方向更明确、重点更突出，对全市经济社会发展产生了重要的指导和推动作用。二是抢抓机遇，乘势而上，加快科学发展的成效更加明显。我们坚持把抓项目和争取政策作为加快发展的头等大事，在应对困难和挑战中捕捉和把握发展机遇，集中实施了一批公共服务建设项目，办成了一批多年来想办的大事实事，发展的基础条件得到明显改观，人民群众得到了更多实惠。三是因地制宜，分类指导，地震灾后恢复重建科学有序推进。我们把灾后重建作为最大的政治、最大的项目和最大的民生工程，坚持从实际出发，坚持分类指导，创造性地提出灾后重建的工作思路和指导原则，精心组织实施灾后重建项目，着力破解重建难题，保证了重建工作有力有效有序开展。四是整合资源，优化配置，加快发展的支撑保障能力明显提升。我们坚持把资源整合作为促进发展方式转变的重要抓手，更加注重推进资源规模化、集约化利用，开展了矿产、土地、项目、资金、人力等资源整合工作，资源配置更趋合理，经济社会发展的质量和效益得到提高，可持续发展的基础进一步夯实。五是以人为本，改善民生，和谐陇南建设迈出新步伐。我们始终坚持把保障和改善民生作为各项工作的出发点和落脚点，在保增长的同时，更加重视发展社会事业和改善民生，着力解决就业、就学、就医、住房和社会保障等群众关心、关注的突出问题，切实做到发展为了人民、发展依靠人民、发展成果由人民共享。六是创新载体，塑造形象，服务全市发展的环境进一步优化。我们坚持把优化环境作为促进发展的基础工程，在不断强化硬环境建设的同时，着力培育和增强软实力，通过集中整治活动和构建长效机制，解决了制约和影响发展的一些突出问题，努力营造科学发展、和谐发展的政策环境、社会环境和干事创业环境。七是市委统揽，各方同心，加快发展的工作合力显著增强。近年来，全市进一步形成了市委坚强领导、人大政协监督支持、政府强力落实的工作格局。各县区党委、政府和各部门、各单位认真贯彻市委、市政府决策部署，创造性地开展工作，驾驭和应对复杂局面的能力、抓落实的执行力明显提高，有力推动了经济社会发展。

各位代表，过去的五年，是我市历史上经济发展最快、基础设施建设变化最大、社会事业发展最好、人民群众得到实惠最多的五年。在罕见的困难和挑战面前，我市经济社会发展取得这样的成绩极其不易。这是党中央、国务院对我们极为关心和支持的结果，是省委、省政府和市委正确领导的结果，是市人大、市政协和社会各界监督支持的结果，是全市人民共同奋斗、开拓进取的结果，是包括深圳人民在内的全国人民大力支持、无私援助的结果。在此，我代表市人民政府向全市各族人民、向驻陇南人民解放军、武警部队致以崇高的敬意！向给予政府工作大力支持的人大代表、政协委员和社会各界人士，向所有为陇南发展作出贡献的朋友们，表示衷心的感谢！

在肯定成绩的同时，我们也清醒地认识到，我市自然条件差、经济总量小、农村贫困面大、自然灾害多的基本市情特征尚未根本改变，经济社会发展面临许多突出矛盾和问题：一是经济结构性矛盾突出，工业化程度低，自我发展能力弱。二是农业基础脆弱，综合生产能力不强，特色农业产业化水平比较低，农民持续增收难度大。三是基础设施欠账多，交通瓶颈制约还比较突出，城镇规模小、功能不完善，城镇化水平低。四是社会事业发展滞后，群众上学难、就医难、行路难问题仍然比较突出，就业和再就业压力大，社会保障水平低，改善民生的任务还很艰巨。五是有些项目建设环境不够宽松，征地拆迁难问题比较突出，项目建设管理水平尚需提高。六是社会转型过程中各类矛盾增多，加强社会管理、维护社会稳定面临不少新情况。七是政府职能转变不到位，服务意识不够强，抓机遇、抓项目的意识和干劲尚需加强，工作作风有待进一步改进。对这些问题，我们将采取有效措施，认真加以解决。

“十二五”时期全市经济社会发展的总体构想

各位代表，市委、市政府对“十二五”时期发展规划高度重视，刚刚召开的市委二届九次全委（扩大）会议通过的《中共陇南市委关于制定国民经济和社会发展第十二个五年规划的建议》，提出了未来五年发展的指导思想、奋斗目标、战略重点和主要任务。根据《建议》精神，市政府制定了《国民经济和社会发展第十二个五年规划纲要（草案）》，已提请大会审议。下面，我就《纲要（草案）》作几点说明。

一、关于“十二五”时期经济社会发展形势的基本判断

“十二五”时期是为2020年全面实现小康社会打好基础的关键时期，是深化改革开放、加快转变经济发展方式的攻坚时期。综合分析和判断国内外、省内外经济社会发展形势，“十二五”时期，我们面临着重大战略机遇和有利条件：一是政策环境比较有利。今年以来国家相继出台了新一轮西部大开发、支持甘肃经济社会发展等一系列政策措施，把陇南列为特殊贫困片带和“两州两市”扶贫攻坚重点区域，为我市加快发展提供了强有力的政策支持。省委省政府提出的“四抓三支撑”总体思路、区域发展战略和跨越式发展思路，必将制定出台支持各地经济社会发展的有利政策，更加有助于我市在经济社会发展上谋求新的突破。二是发展基础得到夯实。我市自身发展正处在一个新的历史起点上，经过改革开放特别是“十一五”以来的奋斗，我市综合经济实力明显增强，社会事业全面进步，人民生活水平稳步提高，特别是通过灾后恢复重建和深圳援建，建成了一批重大基础设施项目，使我市的基础设施面貌发生了前所未有的变化，为“十二五”发展奠定了坚实基础。三是后发优势比较明显。我市自然资源相对比较富集，铅锌、黄金、硅铁等矿产资源丰富，水力资源可开发量约占全省的四分之一，工业、农业特色产业和第三产业发展具有一定规模。特别是“十二五”时期高速公路、铁路和机场等一批交通重大项目的陆续建成，我市交通面貌将发生巨大变化，成为连接西南、西北的重要通道，地理区位优势将得到凸显，为实现跨越式发展创造了有利条件。四是发展活力显著增强。我们在灾后重建和加快科学发展的具体实践形成的总体思路和加快科学发展的机制，顺应了广大干部群众求发展、求和谐的愿望，全市上下形成了心齐气顺、风正劲足、团结协作、争创一流的良好局面。综合分析我市的发展趋势，困难与希望同在，挑战与机遇并存，机遇大于挑战，我市处于可以大有作为的战略机遇期，把实现跨越式发展作为“十二五”时期经济社会的奋斗目标是切实可行的。

二、关于“十二五”规划的指导思想和发展目标

“十二五”时期，我市经济社会发展的指导思想是：坚持以科学发展观为指导，以富民强市为目标，围绕发展与和谐两大主题，抢抓国家深入实施西部大开发战略、支持甘肃经济社会发展等重大历史机遇，实现交通建设、工业发展、城镇建设三大突破，突出特色农业、旅游开发、生态建设、社会事业、改善民生五个重点，强化党的建设、项目带动、优化环境、资源整合和创新机制五项保证，努力推动陇南经济社会跨越式发展。

“十二五”时期，我市经济社会发展的战略目标是：“十二五”时期确保两位数以上的增长速度，到“十二五”末国内生产总值力争翻一番，GDP、工业增加值、固定资产投资、财政收入、城乡居民收入等主要经济指标增长幅度达到或超过全省平均水平。国民经济主要指标实现生产总值年均增长13%以上；规模以上工业增加值年均增长18%以上；全社会固定资产投资年均增长20%以上；财政收入年均增长15%以上；城镇居民人均可支配收入年均增长11%，农民人均纯收入年均增长12%以上；基本建成覆盖城乡的社会保障体系；节能减排实现控制性目标。

“十二五”奋斗目标，主要是根据国内外环境的新变化和全省跨越式发展的新要求，综合考虑了我市发展基础、条件变化和未来发展趋势，既体现了跨越发展的要求，又坚持了实事求是的原则。特别是《纲要（草案）》提出今后五年生产总值年均增长13 %以上的目标，是基于以下考虑：一是当前和长远结合，把“十二五”规划的各项目标与2020年全面建设小康社会的奋斗目标紧密衔接。二是正确把握机遇与挑战，既要抓住机遇，又要正视现实，既体现了规划的战略性、指导性，也留有一定的余地。三是参照全省和其它市州，特别是“两州两市”“十二五”规划指标情况，使“十二五”目标具有横向可对比性。四是针对“十一五”时期我市“5.12”地震灾后重建的特殊性，认真分析了未来五年我市投资、消费、对外贸易等拉动经济增长要素的导向、构成和趋势。

三、关于“十二五”时期的发展重点

《纲要（草案）》对“十二五”时期经济建设、社会发展、改革开放等方面都做出了全面部署，提出了明确的发展重点和主要任务，总体上突出了更加注重以人为本，更加注重全面协调可持续发展，更加注重统筹兼顾，更加注重保障和改善民生。

一是推动特色农业提质增效，夯实社会主义新农村建设基础。坚持把发展特色农业作为调整农村经济结构的主攻方向，根据“尊重规律、扩大规模、强化科技、健全市场、壮大龙头、打造品牌、提质增效”的总体要求，优化特色农业区域布局，立足优势资源条件，努力打造特色农业“四产区、五基地、多片带”发展格局。推进农业产业化进程，推动特色农业规模化发展、标准化生产、商品化加工、市场化营销、科技化支撑。大力推广农业实用技术，提高粮食综合生产能力。大力发展劳务经济，促进农村剩余劳动力输转就业。加强农业基础设施建设，充分利用国家加大水利建设的政策机遇，积极争取项目资金，建设一批江河堤防、农村饮水、渠系灌溉、集雨节灌、水土保持等水利工程。通过努力，在“十二五”末使全市特色产业总面积达到和稳定在1000万亩左右，特色农业产业增加值达到50亿元，努力把陇南打造成为全国优质核桃主产区、全国最大的花椒主产区、全国油橄榄主产区和全省中药材主产区。

二是做大做强工业经济，推动经济结构战略调整。按照“把握大趋势、发展大产业、紧盯大企业、建设大园区、提供大服务”的要求，细化深化工业发展措施，在关键环节进行突破，努力实现工业发展量的扩张和质的飞跃，确保“十二五”末工业增加值实现翻番。加快全市矿产、水力、特色农产品等资源整合步伐，推进企业兼并重组，实现规模化开发和集约式经营，进一步做大做强铅锌、黄金、硅铁、能源、建材、绿色食品、医药化工等工业主导产业。力争使全市铅锌冶炼能力达到30万吨、硅铁冶炼能力达到30万吨、水泥生产能力达到600万吨、白酒生产能力达到3万吨。加快陇南徽县工业集中区、西成开发区和各县工业集中区建设，推动产业集聚发展、企业集中布局、污染集中处理和废弃物循环利用。抓好工业重大项目建设，围绕资源深加工上新项目，力争建成一批支撑工业发展的标志性重大项目，以大项目推动工业大发展。

三是大力发展旅游业，带动第三产业快速增长。积极发展旅游业，加大景区建设力度，完善景区景点配套设施，加快建设精品景区、名牌景区、休闲旅游度假区；打造一批成熟型黄金旅游线路、成长型旅游线路、旅游热线和旅游短线，形成点线结合的精品旅游网络，到“十二五”末，把陇南建成甘陕川毗邻地区生态休闲度假与历史文化观光旅游目的地，使旅游业成为全市的支柱产业。继续提升发展服务业，大力发展商贸流通业，充分利用一批重点交通项目建成带来的契机，新建一批星级宾馆和专业市场、大型商场，建设武都物流中心和陇南商贸重镇，逐步建设各县城物流中心、乡镇配送中心，完善花椒、油橄榄、核桃、木耳、茶叶等特色农产品及中药材集散地交易市场。

四是加强基础设施建设，为经济社会跨越式发展创造条件。坚持重点突破、项目带动的工作思路，着眼强化基础保障，消除瓶颈制约，增强经济社会发展的保障能力。构建综合交通运输体系，统筹铁路、公路、民航和水运建设，构建各种运输方式衔接协调、优势互补、布局合理、运能充分、快捷通畅、安全环保的综合运输网络，把陇南建成连接西南、西北的重要通道。加快高速公路建设，建成武罐高速公路、成武高速公路、十天高速公路陇南段，开工建设渭源至武都高速公路，形成“H”型高速公路网架。抓好农村公路建设，实现市到县、县际出口和联网公路达到二级及以上公路标准，一般公路达到三、四级公路标准。加快兰渝铁路项目建设，实现全线建成通车。积极争取建设煤炭运输通道，推进区域性铁路集运站和枢纽建设。加强城乡电网改造建设，进一步完善以330千伏线路为骨干网架、110千伏线路为主供网架的多电源互供式供电网络，力争每县区至少建设一座110千伏变电站，积极争取建设750千伏输电线路。积极推进通讯网、互联网、电视网“三网”融合发展，着力提高农村和边远地区的信息网络覆盖率，力争行政村基本通宽带，自然村和交通沿线通讯信号基本实现全覆盖。

五是加大城镇建设力度，推进城镇化建设进程。完善城镇规划体系，以陇南市区和各县城为重点，高起点、高标准编制和修编城镇规划。拉大市区和各县县城建设框架，加快推进小城镇建设，优化城镇布局，扩大城镇容量，着力构建以市区为枢纽、以县城为支撑、以中心镇为基础的城镇体系，力争实现全市城镇建成区面积翻一番。抓好道路、通信、供电、供水、供气、供热、停车场、公厕、垃圾和污水处理、园林绿化等城镇基础设施建设和公共服务设施建设，提高宜居水平。加强城镇管理，建立和完善城市建设、土地利用、投资融资、综合执法等机制，推进城镇建设管理制度化、规范化。科学制定房地产开发规划，落实土地经营权招拍挂出让、容积率控制和配套保障房政策，增加住房供应，促进房地产业快速健康发展。逐步放开城镇户籍限制，吸引更多有条件的农村人口入城定居，加快城镇化进程。

六是大力发展社会事业，切实保障和改善民生。把保障和改善民生作为经济社会发展的根本目的，力争在发展社会事业和改善民生方面取得更大进展。坚持优先发展教育，以提高教育质量为核心，统筹各类教育事业的发展。发展卫生事业，完善城市医疗机构功能，加快以城市社区和农村医疗卫生机构为重点的城乡医疗卫生服务体系建设。繁荣城乡文化，加快公益性文化设施建设，实施广播电视村村通和农家书屋等重点文化惠民工程。充分发挥现有科研单位和科技人员的作用，开展技术引进和试验、示范、推广，促进科技成果转化应用。加强人口与计划生育工作，保障困难人群的合法权益。实施人才战略工程，加快人才队伍建设。实施积极的就业政策，鼓励自主创业。加快基本保障体系建设，加快推进新型农村社会养老保险制度，完善城镇居民基本医疗保险制度、新型农村合作医疗制度和城乡医疗救助制度，探索建立城乡一体化的社会保障制度、社会救助和社会福利体系。每年为群众办一批好事实事。

七是努力改善生态环境，促进“两型”社会建设。按照建设资源节约型、环境友好型社会的要求，切实加大生态环境保护和建设力度，增强可持续发展能力。加强白龙江、白水江、西汉水流域及地质灾害隐患区域生态保护与水土流失综合治理，建立健全重大自然灾害监测、预警、预报和应急救援机制，加大城镇防洪体系建设、山洪灾害防治、重点区域地质灾害防治力度，提升防灾减灾能力；积极推进退耕还林、天然林保护和恢复、坡改梯和小流域治理工程，继续实施长江上游水土保持重点防治工程和小水电代燃料项目。坚持走新型工业化道路，围绕“减量化、再利用、资源化”的要求，大力发展循环经济，抓好节能减排工作。强化环境保护，实施以防污减排为重点的环境治理工程，着力解决矿区环境污染、城市大气污染、农村面源污染等突出问题，加强饮水安全保护。

八是不断深化改革，着力提高对外开放水平。加大重点领域和关键环节改革力度，大力培育市场主体，充分发挥市场配置资源的基础性作用。深化行政管理体制改革，促进政府职能转变。深化农村改革，积极稳妥推进土地经营权流转改革，完成集体林权制度改革任务。推进医药卫生体制改革，完善新型农村合作医疗制度。全面推行新型农村社会养老保险制度，推动机关事业单位养老保险制度改革。全面落实扶持非公有制经济发展的政策措施，大力支持非公有制经济发展。创新招商引资方式，完善对外经济交流与合作机制。

2011年的目标任务和工作重点

2011年是实施“十二五”规划的第一年。做好今年的政府工作，要坚持以科学发展观为指导，全面贯彻党的十七届五中全会和全省、全市经济工作会议精神，认真落实市委总体发展思路，进一步解放思想，改革创新，加快转变发展方式，优化经济结构，加强基础设施和生态环境建设，发展壮大特色优势产业，加快社会事业发展，着力保障和改善民生，为“十二五”发展开好局、起好步。

今年经济社会发展的主要预期目标是：生产总值增长13%以上；全社会固定资产投资增长20%以上；大口径财政收入增长14%，市县级财政收入增长11%；工业增加值增长20%，其中规模以上工业增加值增长18%；社会消费品零售总额增长18%；城镇居民人均可支配收入增长11%；农民人均纯收入增长12%；居民消费价格指数控制在3.5%以内；城镇登记失业率控制在4%以内；人口自然增长率控制在6.8‰以内；万元生产总值能耗下降2%左右。

围绕上述目标，着力抓好以下十个方面的工作：

一、进一步抓好灾后重建，着力提升灾区发展水平

坚持不懈地推进灾后恢复重建，奋力夺取灾后重建工作的全面胜利。一是努力完成地震灾后重建任务。坚持分类指导，进一步加大解决征地拆迁等制约项目建设突出问题的力度，加快灾后重建项目建设，力争在“5.12”三周年纪念日之前灾后重建项目竣工率达到95%以上，9月底前力争所有灾后重建项目全部竣工验收并投入使用。落实工作责任，抓紧做好竣工项目验收工作，加快开工项目建设进度，着力解决未开工项目征地拆迁问题，争取尽快开工建设。二是扎实推进“8.12”特大暴洪灾害灾后恢复重建。认真落实支持成县“8.12”特大暴洪灾害灾后重建工作实施方案，切实抓好成县“8.12”灾区重建工作，力争2011年4月底前完成住房维修加固任务，2011年底前完成住房重建任务和60%以上的公共服务、基础设施、地质灾害治理、产业恢复重建任务，为2012年底前全面完成暴洪灾害灾后重建任务打好基础。其它重灾县在利用好省、市已下拨补助资金的同时，采取整合项目资金等办法，切实抓好城乡群众住房、学校、医院和交通重建工作，尽快完成恢复重建任务，力争使灾区基本生产生活条件达到或超过灾前水平。三是促进灾区可持续发展。用足用好财政、税收、土地等灾后重建后续优惠政策，结合“十二五”规划编制，制定全市“5.12”地震灾区发展提升规划，论证一批事关灾后重建成果巩固提升和长远发展的基础设施、产业结构调整、公共服务、生态环境等方面的项目，努力争取国家、省上政策、项目和资金扶持。尽快完善学校、医院等项目的配套设施，研究制定管理办法，提高管理水平，充分发挥项目效益。继续抓好整村重建村的公共服务配套设施建设，抓好村容村貌整治，改善居住环境，将重建村打造成标准较高的社会主义新农村。

二、全力推进项目建设，保持投资稳定增长

充分利用国家西部大开发、支持甘肃经济社会发展等政策措施，实施一批重大项目，增强投资对经济增长的拉动力。一是加快重大项目建设。全力做好协调服务工作，确保兰渝铁路、武罐高速公路、成武高速公路等重大项目完成年度投资计划。开工建设礼县至天水洛门二级公路、礼县至武都县际公路。加快文县临江330千伏变电站、武都东江、洛塘、文县石坊、成县王磨110千伏送变电工程建设进度，新建110千伏西和至长道、西和至礼县、徽县至西和晒经二回线、徽县330千伏变电站，争取尽快建成投入运营。二是加强生态环境保护与建设。以中小河流治理、地质灾害防治为重点，加快重点流域综合治理、山洪灾害防御体系和预警预报体系建设，提高防灾减灾能力。启动“两江一水”生态综合治理工程，继续实施退耕还林、天然林保护、公益林建设、生态修复和自然保护区建设等五大工程，完成退耕还林工程建设配套荒山造林10万亩，实施天然林管护1013.7万亩，重点公益林312.2万亩，完成生态林建设20万亩。三是狠抓项目谋划争取工作。围绕基础设施、特色产业、扶贫开发、生态环境、社会事业、改善民生等国家和省上支持的重点领域，精心谋划一批带动力强、关系陇南长远发展的重大项目，认真做好项目规划论证，增加项目储备，在现有项目库的基础上，筛选储备今年及“十二五”规划重点项目200个以上。加强项目前期工作，重点抓好成州民用机场、十堰至天水高速公路陇南段、“两江一水”等河流水源涵养保护工程和长防林二期工程前期工作，力争今年开工建设；加快渭源至武都高速公路、天水至平凉铁路陇南延伸段项目前期工作，争取早日开工建设。四是加大招商引资力度。建立健全招商引资工作机制，采取节会招商、小分队招商、以商招商等方式，重点抓好工业、特色农业、城市建设、旅游开发等方面的招商引资工作，提高项目签约率和履约率。力争完成签约项目150项以上，到位资金42亿元以上，项目履约率达到80%。

三、切实加强“三农”工作，促进农民收入持续增长

继续加大“三农”投入，全面落实惠农强农政策，促进农业增效、农民增收、农村发展。一是大力发展特色农业产业。大力调整农业结构，不断扩大特色产品基地规模。新发展特色产业面积43.2万亩，其中:核桃14.4万亩、花椒12.7万亩、油橄榄4万亩、中药材1.91万亩、蔬菜2万亩。出栏商品牛羊34.36万头（只），肉蛋奶产量12.79万吨。申报省级龙头企业3户，认定市级龙头企业5户以上。新建农民专业合作社45个，创建专业合作社示范社100个，完成订单农业40万亩。继续抓好注册认证工作，新增无公害农产品产地认定、绿色食品认证、有机食品认证18项，申请注册农产品商标3件，申报国家地理标志保护产品4个以上。创建特色农业示范点109个。二是提高粮食综合生产能力。加强农业科技推广工作，重点抓好良种引进推广、农业综合管理和发展设施农业等工作，完成全膜双垄沟播技术推广18万亩，实现粮食总产98万吨。加强地方粮油储备管理，确保粮食安全。三是深入推进扶贫开发。认真做好扶贫开发和农村低保两项制度衔接工作，积极争取甘肃南部山区特困片带扶贫综合开发试点项目，继续推进整村整片区开发，确保重点县贫困群众人均纯收入年均增幅高于同级平均水平两个百分点。全面落实各项优惠政策，加快民族乡村经济社会发展。加强农民工技能培训，加大农村劳动力输转力度，实现劳务收入57亿元。四是加强农业基础建设。重点抓好礼县苗河水库灌区节水改造等水利工程项目，建设一批小型农田水利设施，年内新增有效灌溉面积3.6万亩、集雨补灌4万亩、节水灌溉4万亩。抓好水土保持工程建设，新修梯田15.88万亩，完成小流域综合治理300平方公里。大力开展植树造林，完成绿色通道建设400公里，义务植树1000万株。推进太阳灶、沼气池等清洁能源和小水电代燃料项目建设，继续抓好农村危房改造和农村环境综合整治工作。

四、加快产业优化升级，推进工业经济聚集发展

按照市委对工业发展的总体要求，以加快转变发展方式为主线，以项目建设为支撑，改造提升传统优势产业，培育新兴产业，加快工业经济发展。一是加快工业项目建设。加快金徽酒业1.5万吨白酒生产线、西和大桥金矿1500吨黄金浮选、西和十里后川坝1500吨黄金浮选、文县100万吨干法水泥等续建项目建设进度，力争年内建成投产；开工建设白银公司成县20万吨锌冶炼系统技术提升、成县10万吨铅冶炼、祁连山集团成县200万吨干法水泥生产线扩建、文县高楼山金矿20万吨黄金浮选、文县临江10万吨特种硅铁、礼县亚特金矿6000吨尾渣回收浮选生产线等项目。二是促进工业经济稳定增长。加强工业经济运行监测分析，及时研究解决存在的困难和问题，确保工业经济健康稳定发展。认真落实扶持优惠政策，重点扶持铅锌、黄金、硅铁、水泥等重点产业、骨干企业正常生产，实现增产增效。加快资源整合步伐，完成厂坝矿区铅锌资源整合后续工作，完善新公司运行机制，促进健康发展；突出抓好管理水平高、核心竞争力强的现代化大企业培育工作，推动产业升级，支撑经济发展。加强市场营销，多方开辟工业产品销售渠道，提高工业经济效益。积极实施品牌战略，重点培育油橄榄、白酒、核桃、茶叶等一批名优特新工业品牌，提高市场竞争力。三是推进工业园区建设。按照产业集聚、项目集中、集约发展的总体要求，规划和建设一批工业园区，构建区域经济结构调整、优化发展布局、转变发展方式的重要平台。重点抓好陇南徽县工业集中区和西成经济开发区两个省级工业园区的基础设施建设，提高项目承载能力，力争在引进项目、承接产业转移上取得实质性进展。抢抓兰渝铁路等重大交通项目建设机遇，积极谋划物流园区和工业园区。四是认真抓好节能减排。科学编制全市循环经济发展规划，论证实施一批资源综合利用和循环经济发展项目。严格落实节能减排目标责任制，加快淘汰小采选、小冶炼、小水泥等落后产能，抓好工业“三废”污染防治、矿区重金属污染和尾矿库治理。

五、加强城镇建设管理，大力提升城镇化水平

着力扩大城市规模，完善城市功能，提升城市品位，大力推进新型城镇化建设。一是建立健全具有陇南特色的规划体系。完成陇南城市规划完善报批，加快各县区县城总体规划修编和供排水、供电、供热、交通、消防、排洪、产业发展等专项规划编制。加快市区及各县区控制性详细规划编制进度，实现县城控制性详细规划全覆盖。抓好重点建设区域、住宅开发建设小区修建性规划编制，强化刚性控制。积极做好重点地段城市景观规划编制，打造城市特色，提升城市品位。加快小城镇规划编制和村镇风貌设计改造，切实解决小城镇规划滞后突出问题。二是加快陇南市区建设。加快基础设施建设，开工建设长江大道东西延伸工程，分别向汉王、两水延伸7.5公里和13.5公里，拉大城市框架，扩大发展空间。加强城市景观风貌设计，抓好南北两山绿化、白龙江水体自然景观建设，精心打造城市亮点，塑造生态人文景观，提升城市形象，把市区建成有一定辐射带动力的山水园林城市，打造成行政中心、商贸中心和交通枢纽。三是加快县城和示范镇建设。加快各县县城路网、管网、供水、供电、垃圾处理等配套基础设施及文县城南堤防、康县城防、宕昌城防等城市防汛工程建设，完善功能，逐步把各个县城打造成特色鲜明、功能完善、服务便捷、环境优美的宜居城镇。加快推进以哈达铺等21个示范小城镇为重点的小城镇建设，改善村镇风貌，提升聚集辐射能力，形成各具特色、布局合理的小城镇体系。四是创新城镇运行和管理机制。合理划分市区两级政府城市管理事权，理顺陇南市区城市管理体制。坚持以治脏、治乱、治差为重点，加强城市市容和环境卫生管理。建立健全城市管理长效机制，完善专业管理和综合执法运行机制，提高城市管理文明执法水平。

六、加快旅游开发，促进第三产业发展

大力发展旅游经济、商贸流通，不断壮大第三产业规模，培育新的经济增长点。一是加快发展旅游产业。抓好全市旅游总体规划和五大精品景区、四大名牌景区修建性详细规划修编，完成市级各类旅游专项规划和宕昌大河坝、武都瑶池天堂、文县白马河休闲旅游度假区修建性详细规划编制工作。加强旅游景区和农家乐管理，搞好旅游从业人员培训，在陇南旅游网站建立武都万象洞、成县西狭颂等重要旅游景区的数字虚拟景区，保持与各知名网站互通链接，为游客提供旅游咨询服务，着力提升旅游形象。加强旅游基础设施建设，论证立项重点旅游景区与高速公路、铁路、交通主干线的连接线路，开通全部精品景区、名牌景区和已开展旅游活动景区移动电话，在市内主要干线公路和城区设置符合国际惯例的旅游交通标牌标识，创建旅游星级饭店5家，提高接待能力。力争全年接待旅游人数达到380万人次，比上年增长18%，旅游综合收入达到10亿元，增长18%。二是努力扩大城乡消费。通过提供丰富的教育文化产品，引导扩大教育、文化消费。以建设大型、高水平批发市场为龙头，推进市场建设，抓好“万村千乡市场工程”、“新网工程”和“集贸市场升级改造”项目建设，加强废旧收购市场管理，新建续建城乡市场37个、农家店和村级综合服务社200个。继续做好“家电下乡”、“家电以旧换新”、“废旧汽车回收”工作。加强房地产市场调控，合理引导住房需求，促进房地产业平稳健康持续发展。

七、做好财税金融工作，努力提高保障能力

充分发挥财政、信贷调节作用，加大对经济社会发展的支持。一是努力增加财政收入。把握国家和省上政策导向，加大力度，积极争取各类财政转移支付。突出强工业、活三产，支持招商引资和工业园区建设，巩固支柱财源，扩大新兴财源，培育后续财源，建立多元化的财源体系。优化财政收入结构，着力提高工业税收比重。坚持依法治税、综合治税，完善税源监控体系，堵塞税收征管漏洞，严厉打击各类偷逃税行为；强化政府非税收入征管，严格执行收支两条线制度，努力提高财政收入占生产总值的比重。二是提高公共财政保障水平。发挥财政把握全局、服务中心的作用，努力提高资金调度管理和支持发展的能力。坚持依法理财、科学理财、民主理财，精打细算、厉行节约，从严控制一般性支出；调整和优化财政支出结构，增加对“三农”、教育、民生工程、生态环境、节能减排、公共安全等方面的投入，支持企业自主创新和新兴产业发展。三是着力改善金融服务。加强对金融工作的指导，建立健全银政、银企沟通协调制度。加强社会信用体系建设，积极支持银行业金融机构争取信贷规模，推进产品创新，扩大信贷投放，破解存贷差持续拉大和企业“贷款难”、银行“难贷款”的问题，实现金融机构自我发展与支持地方经济发展双赢目标。支持保险业发展，不断满足社会保险需求。

八、大力改善公共服务，促进社会事业协调发展

坚持以人为本，统筹经济社会发展，着力保障和改善民生，促进社会全面进步。一是坚持优先发展教育。深入贯彻全市教育工作会议精神，紧紧围绕提高教育质量，加强教师队伍和校长队伍建设，抓好义务教育，巩固“两基”成果，积极发展学前教育、特殊教育，加快推进普通高中课程改革；扎实开展“万名教师培训”工程，落实名教师、名校长岗位津贴，抓好农村教师安居工程建设试点工作。二是加快发展卫生事业。继续完善城乡医疗卫生健康保障体系，实施医疗保障制度；全面实施基本药物制度，在所有政府举办的乡镇卫生院、城市社区服务机构和承担基本公共卫生服务的村卫生室，全面实行国家基本药物目录药品零差率销售；继续完善城乡医疗卫生服务网络，为群众提供安全、有效、方便、价廉的医疗卫生服务。三是积极推动创业带动就业。实施积极的创业就业扶持政策，重点扶持高校毕业生自主创业，以创业带动就业；加强城乡劳动力就业技能培训，完善就业再就业扶持政策，引导劳动者自谋职业和自主创业。全年城镇新增就业1.4万人以上。四是完善社会保障体系。加快市级统筹步伐，做实个人账户，完善城镇居民基本养老和基本医疗、失业、工伤、生育保险制度，积极扩大新型农村社会养老保险试点，努力提高社保经办水平；完善城乡低保、五保户供养、医疗、教育、住房、司法等社会救助和扶老、助残、帮幼社会福利体系，确保低收入群体基本生活；严格执行保障性住房建设问责制度，加快经济适用房、廉租房、公租房建设和棚户区、农村危旧房改造进度，努力改善城乡居民住房条件。五是加强人口工作。全面落实计划生育奖励优先优惠政策，加强流动人口计划生育服务管理，着力解决出生人口性别比失调问题，实施出生缺陷干预，提高出生人口素质，稳定低生育水平。六是加强科技文化广电体育工作。积极培育科技创新示范企业，强化试验示范推广，加快科技成果转化，加强人才队伍建设，深入推进科技特派员创业行动；加快乡镇社区综合文化站、文化大院、农家书屋、广播电视村村通工程建设进度，加强文物和非物质文化遗产保护；加强机关、学校、群众体育工作，广泛开展全民健身活动，重视提高竞技体育水平；加强全民国防教育、国防后备力量建设和人民防空工作，深入开展双拥共建活动；继续做好外事侨务、统计、气象、地震、测绘、档案、地方志等各项工作。

今年，要大力办好改善民生的15件实事。⑴加快扶贫开发，实施扶贫整村推进100个，减少贫困人口8万人。⑵为2万户农民配备太阳灶，新增农村沼气用户1万户。⑶实施饮水安全工程，解决10万农村人口饮水安全问题。⑷实施建制村通畅工程，完成通村水泥路项目51项286.5公里；推进通乡油路建设，完成通乡油路建设项目15项356.7公里。⑸继续实施便民桥工程，解决群众过河难问题。⑹加大对农民工劳务技能培训和组织输转力度，力争全年实施农民工技能培训5万人，劳务输转70万人。⑺提高城乡低保补助标准和水平。城市低保标准提高10%，提高月人均补助水平；提高农村低保补助标准和补助水平。⑻继续实施中小学校舍安全工程，改善城乡办学条件。⑼加快保障性住房建设，新建廉租住房700套、经济适用房717套;给人均住房建筑面积10平方米以下的城市低收入住房困难家庭发放廉租住房租赁补贴；解决5500户城市低收入家庭住房困难。⑽继续抓好城乡危旧房改造工程，实施棚户区改造468户，其中完成林区棚户区改造266户；实施农村贫困残疾人危房改造500户。⑾加快城市社会服务设施建设，在市区和各县城区新建20个停车场，其中市区4个，成县3个，两当1个，其余6县各2个；在市区和各县城区至少建2处水冲式公厕；加强武都区及各县城区供水供电设施建设，解决部分高层住宅楼上水难问题，保障城区电力供应。⑿加强食品安全监管，改造提升食品加工小作坊200户。⒀加强村级卫生室建设；提高新医疗保障水平，提高统筹基金支付限额；乡镇卫生院、城市社区服务机构基本药品零差率销售实现全覆盖；农村基层卫生机构中医药治疗常见病费用纳入新农合门诊统筹资金全额报销工作；为全市400名贫困白内障患者免费实施复明手术。⒁继续推进城乡环境卫生综合整治，加强对40个马路市场的管理；在170个村实施以农村饮用水源保护、生活污水收集处理、生活垃圾收集处理为重点的农村环境综合整治项目，改善农村环境质量；在10个乡镇建设10个垃圾处理场，逐步解决农村生活垃圾无序堆放、污染环境的问题；在武都、成县各建成一处医疗废物集中处置中心，对全市9县区医疗废物等危险废物进行集中处置。⒂兴建4个规模大、水平高的专业市场。

九、深入推进改革开放，不断创新体制机制

加大重点领域和关键环节改革力度，不断推进对外开放，增强发展活力。加快行政管理体制改革，完善市县机构改革，推进乡镇机构改革，推进政府职能转变；深化医药卫生体制改革，全面推进近期五项重点任务，着力抓好基本药物制度建立和公立医院改革试点，为群众提供满意的医疗服务；深化农村综合改革，扩大农村土地流转试点范围，有序推进集体林权配套改革，完成县乡畜牧兽医体制改革任务；加大交通运输关键环节的改革力度；深化财政管理改革，加大部门预算改革力度，积极推进国库集中支付改革，扩大财政支出绩效评价范围。积极发展开放经济，切实加强区域合作，落实与深圳市签订的经济文化合作交流框架协议，实现优势互补、互利双赢；大力调整优化所有制结构，落实促进民营经济和中小企业发展政策，支持非公经济快速发展；出台扶持政策，支持农产品出口基地和出口企业发展，力争完成进出口总额1000万美元。

十、全面加强社会管理，推动和谐陇南建设

不断加强和创新社会管理，着力解决影响社会和谐稳定的突出问题，促进社会和谐。认真贯彻实施《突发事件应对法》，建立和完善应急管理组织体系和应急机制，修订完善专项应急预案，加强政府应急指挥平台和救援能力建设，努力提高应急管理水平。完善信访工作制度，畅通和规范群众需求表达、利益协调、权益保障渠道，及时解决群众合理诉求，积极预防和妥善处置突发性、群体性事件。扎实开展矛盾纠纷排查调处活动，集中力量解决一批群众反映强烈的热点难点问题。严格落实安全生产责任制，突出抓好交通运输、矿山、消防、危险化学品、烟花爆竹、旅游景区、水电开发、建筑和桥梁隧道施工、公共场所等事故多发领域的专项整治，全面排查治理安全隐患，严防重特大事故发生。整顿和规范市场秩序，强化产品质量和食品药品安全监管，保护消费者合法权益。加强物价监测调控，保障粮食、蔬菜等生活必需品的供应，保持市场物价基本稳定。加强社会治安综合治理，深入推进社会矛盾化解、社会管理创新和社会治安防控体系建设，依法严厉打击违法犯罪，解决好影响公众安全感的突出治安问题，保障人民生命财产安全。大力开展法制宣传教育，做好法律援助和社区矫正工作。

各位代表，谋划好“十二五”发展，全面完成2011年各项任务，使命光荣，责任重大。我们将不断加强政府自身建设，奋力进取，真抓实干，更好地对人民负责、为人民服务、让人民满意。一是着力推进依法行政。认真贯彻落实国务院《关于加强法治政府建设的意见》精神，牢固树立法治理念，依照法定权限和程序管理经济社会事务。自觉接受市人大的依法监督、市政协的民主监督、社会公众和新闻舆论监督，认真听取各民主党派、工商联、无党派人士的意见建议。完善重大事项公示、听证、专家咨询和政府新闻发布等制度，努力提高依法、科学、民主决策水平。强化执法责任，认真做好行政复议和行政应诉工作，严格执行规范性文件备案审查制度，认真履行政府承诺，让人民赋予的权力在阳光下运行，努力提升政府公信力。二是着力优化政务服务。继续坚持优化发展环境，把经济工作的重点转移到为市场主体服务上来，健全完善长效机制，增强服务意识，转变服务态度，改进服务作风，提高服务群众、服务企业、服务基层的能力和水平。完善政府机关服务群众的便民措施，加强行政服务中心、政务大厅和电子政务建设，把与企业、社会和人民群众密切相关的行政管理事项都纳入政务大厅办理，健全公开办事制度，全面推行服务承诺、首问负责、限时办结等制度，完善一站式审批、一条龙服务，着力打造便民高效的政务服务环境。三是着力提高行政效能。加强对省委、省政府和市委、市政府重大决策部署执行情况的监督检查，确保政令畅通。深入推进政务公开和政府信息公开，继续推进行政审批制度改革，减少行政许可和审批事项。建立科学合理的政绩考核机制，严格落实重点工作目标责任考核，继续实行市政府与各县区、各部门签订重点目标任务专项工作责任书，着眼实效，狠抓关键环节，加大督查督办力度，年终集中统一考核奖评，严格兑现奖罚。加强对重点项目、重点产业、重点工作的督查督办，着力解决管理不到位、措施不到位、落实不到位和群众不满意的问题，决定了的事情要及时启动，部署了的工作要一抓到底，坚决纠正有令不行、有禁不止、推诿扯皮等不良作风。大力精简会议、文件和事务性活动，把更多时间、精力用到抓工作落实上来。四是着力加强作风建设。始终坚持把改进工作作风、狠抓工作落实作为加强政府自身建设的重要内容，切实提高公共服务、社会管理和群众工作的水平。健全完善目标责任制和抓落实的工作机制，继续实行领导干部包抓项目、包抓产业制度和联系县区、乡村、企业、学校及创办示范点的制度。深入推进创先争优活动，加强与基层群众的联系，夯实基层工作基础。坚持从实际出发，大兴调查研究之风，着力解决资金筹措、征地拆迁、项目审批等问题，用最大的精力、最有效的方法，全力推进，力求实效。坚持一切从实际出发，察实情、出实招、办实事，不搞形象工程，不做表面文章，力求做到言必责实、行必责实、功必责实。五是着力加强廉政建设。坚持用制度管权、管事、管人，认真落实党风廉政建设责任制，扎实推进惩治和预防腐败体系建设，严格执行《廉政准则》，深入开展反腐倡廉警示教育活动，不断完善权力运行监控机制。加强行政监察和财政、审计监督，强化对重点领域、重点行业的监督，重点抓好灾后重建资金、扩大内需资金、政府投资项目资金的监管和审计，切实抓好查出问题的整改落实。深入开展工程建设领域突出问题和“小金库”专项治理，坚决治理和纠正损害群众利益的突出问题和不正之风，严肃查处各类违法违纪案件，保证人民赋予的权力始终用于为人民谋利益。

各位代表，我们正站在新的起点上，时代使命赋予重托，崛起钟声催人奋进。让我们在省委、省政府和市委的坚强领导下，在市人大、市政协和全市人民的监督支持下，锐意进取，开拓创新，奋发图强，扎实工作，为实现陇南经济社会跨越式发展而努力奋斗！
